# Supplementary material for: Chemical, physical, and functional properties of Thai indigenous brown rice flours
Source: PLoS One. 2021 Aug 3;16(8):e0255694. doi: 10.1371/journal.pone.0255694 (PMC8330919; doi:10.1371/journal.pone.0255694)
Supplement: Attachment — Submitted filename: comments PLOS.docx [file pone.0255694.s001.docx]

**Comments to the Author**

The manuscript is focused on the chemical, physical, and functional properties of Thai indigenous brown rice flour in comparison with JMRF) and CMRF. Overall, the manuscript is well designed and well written. However, the manuscript needs further improvement before acceptance. The authors should pay attention to the following specific points.

**Specific comments:**

1. The Abstract should be a bit more concise and needs further improvement.
2. Thoroughly check the manuscript for grammatical and syntax errors. Some sentences need to be paraphrased with proper scientific wordings.
3. Line 71: Change “which is” to “which are”
4. Line 90: Change “gain” to “get”
5. Lines 102,103: The sentence needs improvement
6. Lines 146, 147: The two sentences should be merged together. Write it as “….with gentle stirring and then centrifuged at …..”
7. Lines 181, 182: The sentence needs to be improved.
8. Line 186: “were reported”?. It is not very clear and need to be paraphrased properly. Such as “…. Setback were measured according to the method of…”
9. Line 188: Should be “…. And the experiments were performed in triplicate”.
10. The subtitles of the Results section should be written based on the results of your study.
11. Line 196: Add comma before “whereas”
12. Line 200: Remove comma after “KFRF/JMRF,”
13. Line 269: Add comma before “as shown in Table 3.”
14. Line 271: Add comma before “as illustrated in Table 3.”
15. No need to give subtopics in the Discussion section
16. Lines 290, 291: The sentence needs to be paraphrased.
17. The discussion section is too long and needs to be properly revised and should be written a bit brief. Moreover, there are some minor syntax and grammatical errors, which should be carefully checked.
18. In the conclusion, write about the significance of your study. What benefits it could provide to the industry.
